# Oncolytic virus synergizes with Smac mimetic compounds to induce rhabdomyosarcoma cell death in a syngeneic murine model

**DOI:** 10.18632/oncotarget.13849

**Published:** 2016-12-10

**Authors:** Christine C Dobson, Thet Naing, Shawn T Beug, Mame D Faye, Janelle Chabot, Martin St-Jean, Danielle E Walker, Eric C LaCasse, David F Stojdl, Robert G Korneluk, Martin Holcik

**Affiliations:** ^1^ Molecular Biomedicine Program, Apoptosis Research Centre, Children's Hospital of Eastern Ontario Research Institute, Ottawa, ON, Canada; ^2^ Department of Cellular and Molecular Medicine, University of Ottawa, Ottawa, ON, Canada; ^3^ Department of Biochemistry, Microbiology and Immunology, University of Ottawa, Ottawa, ON, Canada; ^4^ Department of Pediatrics, University of Ottawa, Ottawa, ON, Canada

**Keywords:** rhabdomyosarcoma, Smac mimetic, oncolytic virus, immunotherapy

## Abstract

Rhabdomyosarcoma (RMS), a neoplasm characterized by undifferentiated myoblasts, is the most common soft tissue tumour in children. Therapeutic resistance is common in RMS and is often caused by acquired defects in the cellular apoptotic program. Smac mimetic compounds (SMCs) are a novel class of inhibitor of apoptosis (IAP) antagonists that are currently under clinical development as cancer therapeutics. We previously reported that cIAP1 is overexpressed in human primary RMS tumours and in patient-derived RMS cell lines where it drives resistance to apoptosis. In this study, we investigated whether inflammatory cytokine production triggered by activators of innate immunity synergizes with LCL161 to induce bystander killing of RMS cells *in vitro* and *in vivo*. Indeed, we show that innate immune stimuli (oncolytic virus (VSVΔ51-GFP), interferon γ (IFNγ), and tumour necrosis factor-like weak inducer of apoptosis (TWEAK)) combine with SMCs *in vitro* to reduce cell viability in the Kym-1 RMS cancer cell line. Other human RMS cell lines (RH36, RH41, RD, RH18, RH28, and RH30) and the murine RMS cell line 76-9 are resistant to treatment with LCL161 alone or in combination with immune stimulants in *in vitro* cell viability assays. In contrast, we report that the combination of LCL161 and VSVΔ51-GFP reduces tumour volume and prolongs survival in a 76-9 syngeneic murine model. Our results support further exploration of the combined use of IAP antagonists and innate immune stimuli as a therapeutic approach for RMS cancers.

## INTRODUCTION

Rhabdomyosarcoma (RMS) is the most common soft tissue sarcoma in children, representing 3-4% of all childhood cancers [1]. RMS is a malignant neoplasm of presumed mesenchymal origin and is characterized by undifferentiated myoblast-like cells [2, 3]. The two major diagnostic subtypes of RMS, embryonal RMS (ERMS) and alveolar RMS (ARMS), differ based on genetic signature, age of onset, primary tumour site, invasion and metastatic risk, and overall survival [4, 5]. When treated aggressively, patients have a high long-term survival rate; however, therapeutic resistance is common in cases of recurrent and/or metastatic RMS [4, 5]. Therefore, there is a clear need to better understand the molecular basis of therapeutic resistance in RMS, which may identify new and effective treatment options that will improve long-term outcomes for patients.

The failure of current chemotherapeutic approaches to eradicate tumour cells is frequently associated with defects in the cellular apoptotic program. In fact, impaired apoptosis plays a key role in the pathogenesis of RMS and contributes to chemotherapeutic resistance by two main mechanisms: 1) preventing cells from responding to pro-apoptotic signals and 2) via the upregulation of anti-apoptotic proteins [6]. In particular, the inhibitor of apoptosis (IAP) protein family are critical regulators of apoptosis and are frequently overexpressed in human cancers, often with transforming or tumour-supporting properties [7]. The eight human IAP members are defined by the presence of a canonical zinc-finger BIR domain and a carboxy-terminal RING finger domain with E3 ubiquitin ligase activity [8]. Among this family of proteins, the structurally similar cellular IAP 1 (cIAP1) and cIAP2 proteins are key regulators of tumour necrosis factor-α (TNFα) and related cytokine members signaling through regulation of the classical and alternative nuclear factor-κB signaling pathways [8, 9]. cIAP1 and cIAP2 are also important regulators of the extrinsic apoptosis pathway and act to control caspase-8 medicated cell death [8]. In addition, X-linked IAP (XIAP) can impact TNFα signaling and NF-κB activation through its inhibition of caspase-3 and caspase-7 and has been shown to suppress TNF-related apoptosis inducing ligand (TRAIL)-induced cell death [8, 10]. Since the IAP proteins play a role in human cancer progression, they have long been considered a promising target for therapeutic intervention [7]. Indeed, Smac mimetic compounds (SMCs, e.g. LCL161) based on the endogenous IAP antagonist termed Smac, promote apoptosis by causing ubiquitin-mediated proteasomal degradation of cIAP1 and cIAP2, thereby enhancing caspase activation [8]. In general, tumour cell lines that can produce endogenous TNFα (~5-10% of cancer cell lines) are sensitive to SMCs alone, but pro-death cytokine ligands are often required for maximal SMC efficacy [8, 11, 12].

Recently, we reported that the insulin-like growth factor 2 mRNA-binding protein 1 (IGF2BP1) is overexpressed in both ERMS and ARMS patient-derived cell lines and in primary RMS tumours [13]. Indeed, we determined that IGF2BP1 binds cIAP1 mRNA and mediates its translation *via* the 5’-untranslated region (UTR) internal ribosome entry site (IRES). We further demonstrated that reducing the levels of cIAP1 either by IGF2BP1 knockdown or by treatment with LCL161 sensitized RMS cell lines to TNFα-mediated cell death. Finally, we tested this approach *in vivo* in a xenograft mouse model using the human ERMS cell line Kym-1, which has autocrine TNFα production and is therefore sensitive to SMC treatment as a single agent [14]. Indeed, SMC treatment inhibited the establishment and growth of Kym-1 xenograft tumours and extended survival in mice. However, most RMS do not produce endogenous TNFα and initial *in vitro* testing has suggested that the human RMS cell lines RD, RH41, RH30, and RH18 are resistant to treatment with LCL161 [15]. Furthermore, LCL161 treatment did not inhibit tumour growth in six RMS xenograft tumours when used as a single agent [15]. SMCs have proven to be safe and well tolerated in phase 1 and phase 2 clinical trials, but have limited efficacy in highly refractory and relapsed cancer patient populations [8]. This evidence suggests that SMCs will require other pro-death cytokine ligands to effectively treat most RMS cancers. We recently demonstrated that SMCs synergize with innate immune stimuli, including oncolytic viruses and recombinant interferon, to induce an effective and safe cytokine storm that promotes tumour death in murine models of breast and colon carcinomas [16]. We hypothesize that this combined treatment paradigm will also promote cell death in RMS cancers.

Here, we report that the human RMS cell line Kym-1 is sensitive to LCL161 as a single agent, while other human RMS cell lines (RH36, RH41, RD, RH18, RH28, and RH30) and the murine RMS cell line 76-9 are resistant to LCL161 as a single agent. The resistance of these cell lines does not appear to be related to alterations in apoptosis pathway effectors or in immune modulator receptors. Importantly, innate immune stimuli (e.g. oncolytic virus (VSVΔ51-GFP), interferon γ (IFNγ), and tumour necrosis factor-like weak inducer of apoptosis (TWEAK)) synergize with LCL161 *in vitro* to promote TNFα signaling and reduce cell viability in Kym-1 RMS cancer cells. In contrast, *in vitro* cell viability assays showed that all other RMS cell lines tested were also resistant to combined treatment with LCL161 and immune stimulants. Importantly, targeting the IAPs and stimulating cytokine signaling in an *in vivo* 76-9 syngeneic model using LCL161 and VSVΔ51-GFP resulted in reduced tumour volume and durable cures in mice. Our results advocate for the combined use of IAP antagonists and innate immune stimuli as a potential therapeutic approach for RMS cancers.

## RESULTS

### Kym-1 cells, but not other RMS cell lines, are sensitive to LCL161 as a single agent

**Figure 1:**
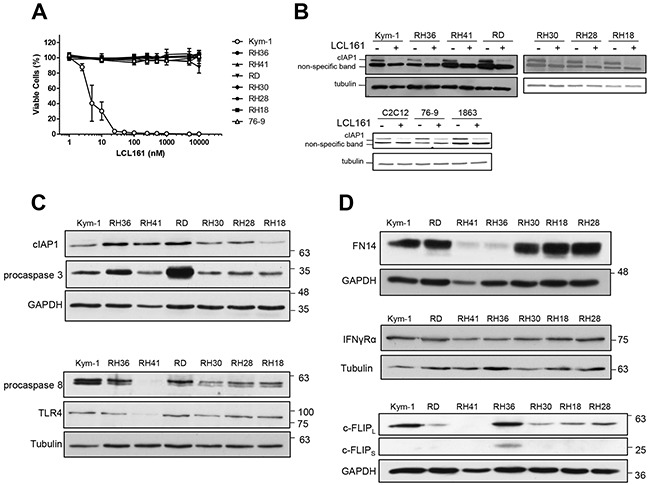
Kym-1 cells, but not other RMS cell lines, are sensitive to LCL161 as a single agent **A**. Alamar Blue viability assays of human RMS cell lines (Kym-1, RH36, RH41, RD, RH30, RH28, RH18) and the murine cell line 76-9 with increasing concentration of LCL161 for 24 h. **B**. Human RMS cell lines were analysed by western blot for cIAP1 and tubulin protein expression following treatment with vehicle control (DMSO) or 5 μM LCL161 (10 nM LCL161 was used for Kym-1 cells) for 24 h. The mouse C2C12 myoblast, 76-9 RMS, and 1863 sarcoma cell lines were also analysed by western blot for cIAP1and tubulin protein expression following treatment with DMSO or 5 μM LCL161 for 24 h. **C, D**. Endogenous protein expression of apoptosis pathway effectors and immune modulator receptors was analysed by western blot in human RMS cell lines (C and D, respectively).

The human ERMS cell line Kym-1 was highly sensitive to exposure to increasing concentrations of LCL161 for 24 h (Figure 1A, open circles). Viability of Kym-1 cells was assessed by Alamar Blue assay and was significantly reduced to 40.48%, 30.28%, and 3.80% following 24 h incubation with media containing 5 nM, 10 nM, and 25 nM of LCL161, respectively. When Kym-1 cells were treated with concentrations of ≤100 nM of LCL161 for 24 h, cell viability was reduced to levels that were indistinguishable from blanks (i.e. samples containing media and Alamar Blue reagent, but no cells). In contrast, concentrations of LCL161 up to 10 M had no effect on viability in all other human RMS cell lines (RH36, RH41, RD, RH30, RH28, and RH18) and in the mouse cell line 76-9 (Figure 1A). To determine whether sensitivity of RMS cells to LCL161 was related to cIAP1 protein expression, western blotting was used to assess cIAP1 expression in cells treated with vehicle (DMSO) or LCL161 for 24 h (Figure 1B). Treatment with 5 μM LCL161 (10 nM LCL161 in Kym-1 cells) for 24 h resulted in similar reductions in cIAP1 protein expression in all human RMS cell lines tested, and therefore was not associated with LCL161 sensitivity. Similarly, treatment with 5 μM LCL161 resulted in reduced cIAP1 protein expression in mouse C2C12 myoblast, 76-9 RMS, and 1863 sarcoma cell lines. Of note, the expression of XIAP was unaffected by LCL161 treatment (Supplementary Figure S1) while cIAP2 is not expressed in cells of muscle lineage [13]. Next, endogenous protein expression of apoptosis pathway effectors was analysed by western blot in human RMS cell lines (Figure 1C). There were no consistent differences in expression of cIAP1, procaspase-3, procaspase-8, or TLR4 in LCL161-resistant RMS cell lines as compared with Kym-1 cells, which are highly sensitive to LCL161. Of note, RH41 cells had reduced expression of procaspase-8, which may be related to apoptotic resistance. Protein expression of immune modulator receptors was also analysed by western blot to determine whether LCL161-resistant cell lines were deficient in pro-death cytokine signaling pathway effectors (Figure 1D). FN14 expression was reduced in RH41 and RH36 cell lines, but not in other LCL161-resistant RMS cell lines. Furthermore, there was no correlation between IFNγRα or cellular FLICE (FADD-like IL-1β-converting enzyme)-inhibitory protein (c-FLIP) protein expression and LCL161 sensitivity of RMS cell lines.

### LCL161 and innate immune stimuli synergize to reduce viability in Kym-1 cells

**Figure 2:**
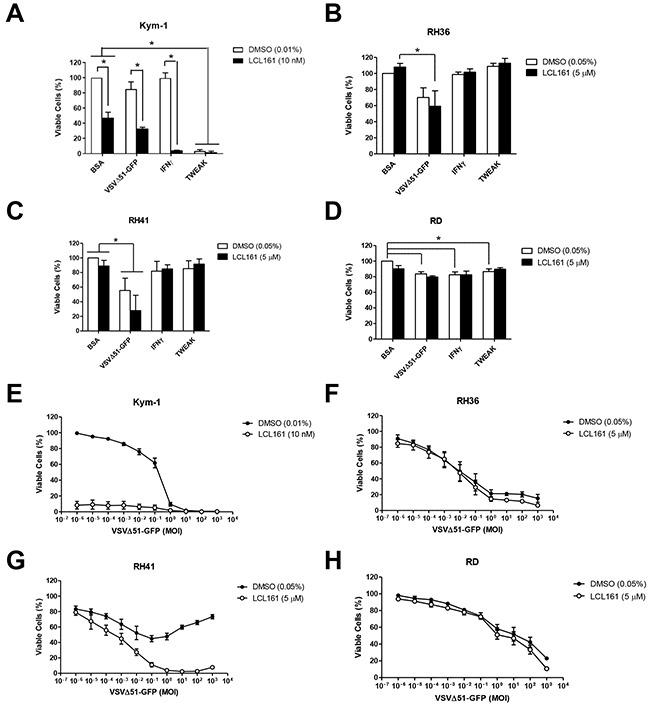
Sensitivity of RMS cell lines to innate immune stimuli in the presence of LCL161 **A**. Kym-1 cells were treated with vehicle control (DMSO) or 10 nM LCL161 and the following innate immune stimulants: BSA control (100 ng/mL), VSVΔ51-GFP (MOI 0.1), IFNγ (1000 U/mL), or TWEAK (100 ng/mL). **B, C, D**. Alamar Blue viability assays were performed after 24 h. LCL161-resistant RMS cell lines (RH36, RH41, and RD) were treated with DMSO or 5 μM LCL161 and innate immune stimulants at the same concentrations as stated above, followed by Alamar Blue viability assays after 24 h (B, C, and D, respectively). **E**. Alamar blue viability assays of Kym-1 cells treated with vehicle or 10 nM LCL161 and increasing MOI of VSVΔ51-GFP for 24 h. **F-H**. RH36, RH41, and RD cells were treated with vehicle or 5 μM LCL161 and increasing MOI of VSVΔ51-GFP for 24 h, followed by Alamar blue viability assays. (*,*p* < 0.05).

In order to determine the sensitivity of RMS cells to a combined treatment paradigm, cell lines were treated with vehicle control (DMSO) or 5 μM LCL161 (10 nM LCL161 in Kym-1 cells) in the presence of the following innate immune stimuli: VSVΔ51-GFP, IFNγ, TWEAK, or bovine serum albumin (BSA) as a control (Figure 2). Cell viability was assessed after 24 h by Alamar Blue assay (Figure 2) and YOYO-1 staining (Supplementary Figure S2) while caspase 3/7 activation was measured by IncuCyte live cell kinetic imaging (Supplementary Figure S2). Interestingly, VSVΔ51-GFP (multiplicity of infection (MOI) 0.1) and IFNγ (1000 U/mL) were not effective when used as a single agent, but both agents synergized with LCL161 to significantly reduce viability in Kym-1 cells (Figure 2A). In addition, Kym-1 cells were highly sensitive to treatment with 100 ng/mL TWEAK, regardless whether it was used in combination with LCL161. In comparison, IFNγ and TWEAK did not reduce viability in RH36 or RH41 cell lines, nor did the immune stimulants synergize with LCL161 (Figure 2B-2D). The addition of VSVΔ51-GFP resulted in significantly reduced cell viability in LCL161-treated RH36 cells. Furthermore, VSVΔ51-GFP decreased cell viability in both DMSO-treated and LCL161-treated RH41 cells. Cell viability was reduced in DMSO-treated RD cells with the addition of VSVΔ51-GFP, IFNγ, and TWEAK. LCL161 did not synergize with immune stimulants in RD cells to promote cell death. Since VSVΔ51-GFP treatment promoted at least a modest reduction in viability in all RMS cell lines tested, we further assessed the effect of oncolytic virus on cell viability in these cells. We performed Alamar Blue viability assays of RMS cell lines treated with vehicle or LCL161 and increasing MOI of VSVΔ51-GFP for 24 h (Figure 2E-2H). Indeed, while all four RMS cell lines were sensitive to VSVΔ51-GFP, synergistic reductions in cell viability were only observed in Kym-1 and RH41 cells that were treated with LCL161. Of note, the long term survival (as determined by a clonogenic assay) was severely impacted in all four cell lines treated with VSVΔ51-GFP with or without LCL161 (Supplementary Figure S3).

### TNFα and IRF1 mRNA expression are increased in RMS cell lines after treatment with innate immune stimuli

**Figure 3:**
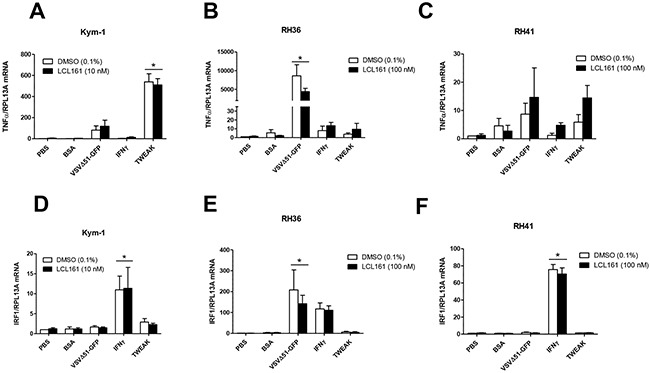
TNFα and IRF1 mRNA expression in RMS cell lines after treatment with innate immune stimuli **A, D**. Kym-1 cells were treated with vehicle control (DMSO) or 10 nM LCL161 and the following innate immune stimuli: PBS control, BSA control (80 pg/mL), VSVΔ51-GFP (MOI 0.1), IFNγ (1000 U/mL), TWEAK (100 ng/mL) and mRNA expression of TNFα and interferon regulatory factor-1 (IRF-1) was assessed by qRT-PCR. LCL161-resistant RMS cell lines (RH36 and RH41) were treated with DMSO or 100 nM LCL161 and PBS, BSA, VSVΔ51-GFP, IFNγ, and TWEAK for 24 h, followed by qRT-PCR analysis of TNFα (**B, C**.) and IRF-1 (**E, F**.) mRNA expression.

We evaluated whether the resistance of RMS cells to LCL161 and innate immune stimuli was related to impaired mRNA expression of known downstream targets of the NF-κB and/or immunomodulatory signaling pathways. We examined mRNA expression of TNFα and interferon regulatory factor 1 (IRF1) in Kym-1, RH36, and RH41 cells following treatment with vehicle control (DMSO) or LCL161 and the following innate immune stimuli: VSVΔ51-GFP, IFNγ, TWEAK, BSA, or phosphate buffered saline (PBS) as a control (Figure 3; Supplementary Figure S4)). IRF1 is a known regulator of type I IFN expression and is responsive to IFNγ and viral infection [17, 18]. Treatment of Kym-1 cells with 10 nM LCL161as a single agent did not affect TNFα mRNA expression in PBS- or BSA-treated cells (Figure 3A). Furthermore, TNFα mRNA expression in Kym-1 cells was unaffected by IFNγ (1000 U/mL) when used alone or in combination with LCL161. In contrast, TNFα mRNA expression increased >100-fold and >500-fold in Kym-1 cells treated with VSVΔ51-GFP (MOI 0.1) and TWEAK (100 ng/mL), regardless of whether it was used alone or in combination with LCL161. In RH36 cells, VSVΔ51-GFP treatment combined with DMSO or 100 nM LCL161 resulted in a >8000-fold and >4000-fold increase in TNFα mRNA, respectively (Figure 3B). TNFα mRNA levels in RH36 cells were unaffected by treatment with IFNγ or TWEAK compared with PBS-treated cells. In RH41 cells, there were no statistically significant changes in TNFα mRNA expression after treatment with DMSO or LCL161 in combination with VSVΔ51-GFP, IFNγ, or TWEAK (Figure 3C). IRF1 mRNA expression was significantly increased in Kym-1 cells following treatment with IFNγ and either DMSO or LCL161 (Figure 3D). VSVΔ51-GFP treatment resulted in a >100-fold induction in IRF1 mRNA expression in both DMSO-treated and LCL161-treated in RH36 cells, but there was no statistical discernible effect of the other immune stimulants on these cells (Figure 3E). Finally, IFNγ increased IRF-1 mRNA expression >70-fold in both DMSO-treated and LCL161-treated RH41 cells (Figure 3F). IRF-1 mRNA levels were not affected by other immune stimulants in RH41 cells.

### LCL161 and innate immune stimuli do not affect viability of murine 76-9 RMS cells *in vitro*

**Figure 4:**
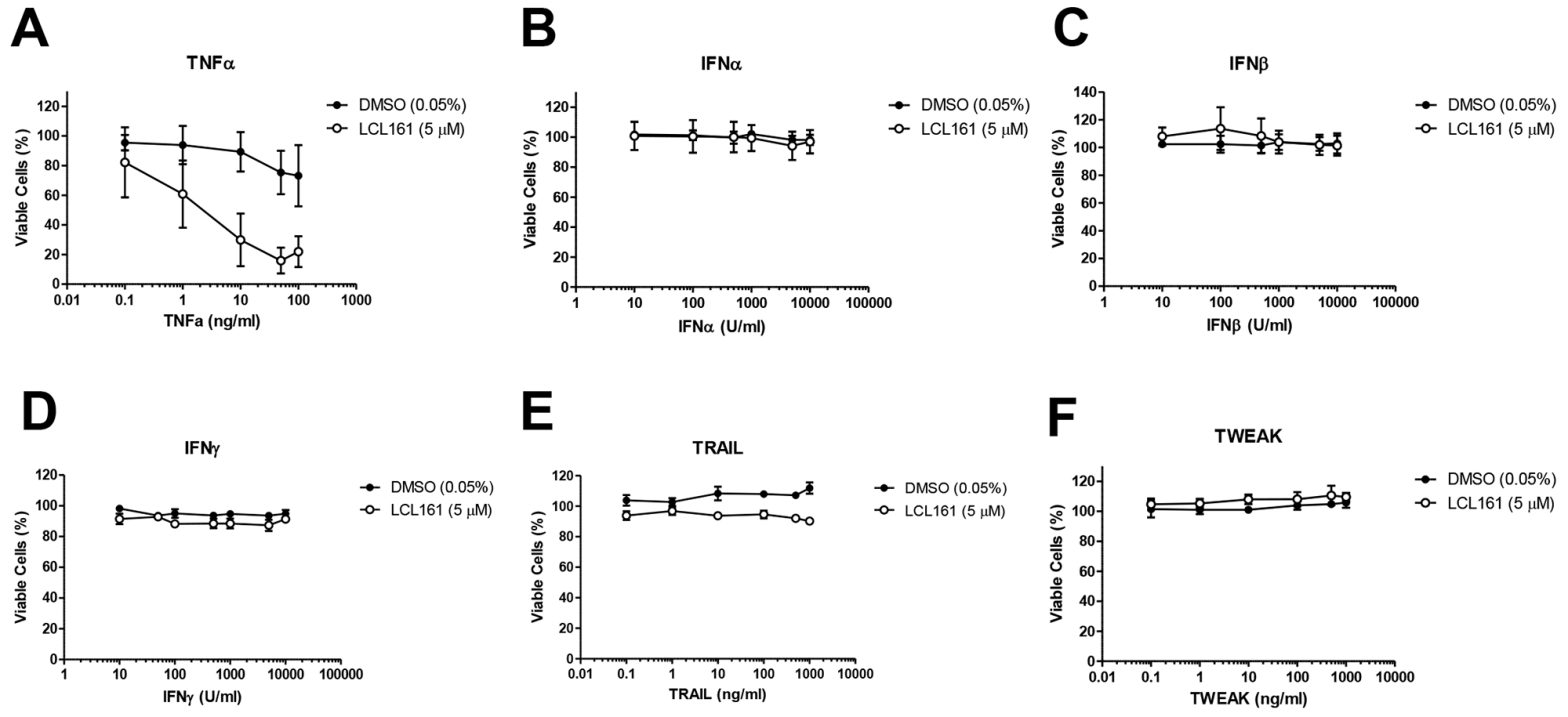
LCL161 and innate immune stimuli do not affect viability of 76-9 RMS cells in vitro **A**. Murine 76-9 RMS cells were treated with vehicle control (DMSO) or 5 μM LCL161 and increasing concentrations of the following innate immune stimulants: TNFα (positive control), universal type I IFNα, IFNβ, IFNγ, TRAIL, and TWEAK.

To test the efficacy of combined LCL161 and innate immune stimuli in the murine 76-9 RMS cell line, cells were treated with vehicle control (DMSO) or 5 μM LCL161 in the presence of increasing concentrations of the following innate immune stimuli: TNFα (positive control), universal type I IFNα, IFNβ, IFNγ, TRAIL, and TWEAK (Figure 4). Cell viability was assessed after 24 h by Alamar Blue assay. We observed that LCL161 sensitized 76-9 cells to TNFα-mediated cell death (Figure 4A). Viability of 76-9 cells was reduced to 15.92% following 24 h treatment with LCL161 and 50 ng/mL TNFα as compared with cells treated with DMSO and 50 ng/mL TNFα, which were 75.38% viable (*p* < 0.05). In contrast, viability of 76-9 cells was unaffected by universal type I IFNα, IFNβ, IFNγ, TRAIL, and TWEAK. Furthermore, LCL161 did not combine with any of these innate immune stimuli to reduce viability in 76-9 cells (Figure 4B-4F).

### Oncolytic virus (VSVΔ51-GFP) reduces viability of 76-9 cells *in vitro*, but does not synergize with LCL161

**Figure 5:**
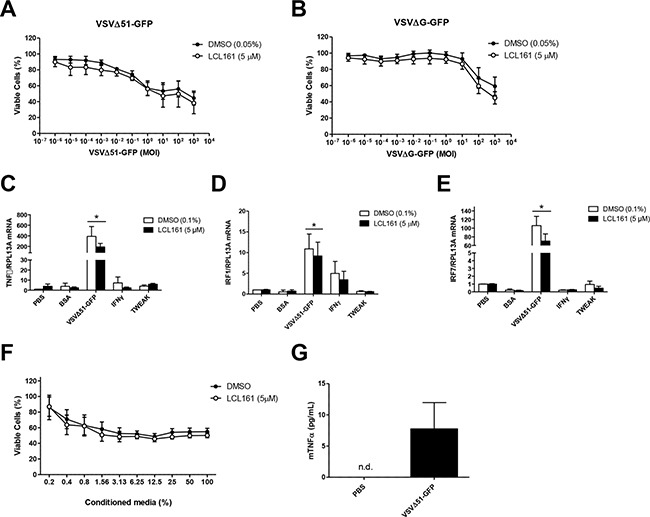
VSVΔ51-GFP reduces viability of 76-9 cells in vitro, but does not synergize with LCL161 **A, B**. 76-9 RMS cells were treated with vehicle (DMSO) or 5 μM LCL161 and increasing MOI of VSVΔ51-GFP or the non-replicating VSVΔG-GFP. **C-E**. 76-9 cells were treated with vehicle control (DMSO) or 5 μM LCL161 and innate immune stimuli, including BSA (80 pg/mL), PBS control, VSVΔ51-GFP (MOI 0.1), IFNγ (1000 U/mL), TWEAK (100 ng/mL). mRNA expression of TNFα, interferon regulatory factor 1 (IRF1) and IRF7 was assessed by qRT-PCR. **F**. Cell culture supernatant from VSVΔ51-GFP-infected (MOI 0.01) 76-9 cells was UV inactivated and applied to naïve 76-9 cells in the presence of DMSO or 5 μM LCL161. **G**. TNFα concentration in media from 76-9 cells after treatment with PBS or VSVΔ51-GFP (MOI 0.01) was measured by using ELISA.

In order to further evaluate the cytotoxicity of VSVΔ51-GFP in the murine 76-9 RMS cells, we performed Alamar Blue viability assays after 24 h treatment with vehicle (DMSO) or 5 μM LCL161 and increasing MOI of VSVΔ51-GFP or the non-replicating VSVΔG-GFP (Figure 5A and 5B, respectively). 76-9 cells were sensitive to VSVΔ51-GFP, with a ~40% reduction in cell viability at an MOI of 1, but LCL161 did not synergize with the virus to further promote cell death (Figure 5A). 76-9 cells were not sensitive to VSVΔG-GFP at biologically relevant MOI, which indicates that viral replication is required to reduce viability in these cells (Figure 5B). We also examined mRNA expression of TNFα, IRF1, and IRF7 in 76-9 cells following treatment with vehicle control (DMSO) or 5 μM LCL161 and the following innate immune stimuli: VSVΔ51-GFP (MOI 0.1), IFNγ (500 U/mL), TWEAK (100 ng/mL), BSA (80 pg/mL) and (PBS) as a control (Figure 5C-5E). VSVΔ51-GFP combined with DMSO or LCL161 resulted in significantly increased mRNA expression of TNFα (>190-fold increase), IRF1 (>9-fold increase), and IRF7 (>70-fold increase). Treatment with IFNγ or TWEAK did not alter TNFα, IRF1, or IRF7 mRNA expression compared with PBS-treated controls. To test whether treatment with VSVΔ51-GFP results in the production of secreted cytokines in 76-9 cells that are cytotoxic to neighbouring cells, cell culture supernatant from VSVΔ51-GFP-infected 76-9 cells was viral inactivated and applied to naïve 76-9 cells in the presence of DMSO or 5 μM LCL161 (Figure 5F). Increasing the concentration of conditioned media resulted in a significant reduction in viability in naïve 76-9 cells, whereby cells treated with 100% conditioned media were <60% viable. Finally, we tested whether TNFα was secreted from 76-9 cells after treatment with LCL161 and oncolytic virus using enzyme-linked immunosorbent assay (ELISA) (Figure 5G). TNFα concentration was measured in media from 76-9 cells treated with PBS or VSVΔ51-GFP (MOI 0.01). There was no detectable TNFα in media from cells treated with PBS, but treatment with VSVΔ51-GFP resulted in ~7.5 pg/mL TNFα in media.

### Combination treatment with LCL161 and VSVΔ51-GFP inhibits tumour growth in 76-9 syngeneic mice

**Figure 6:**
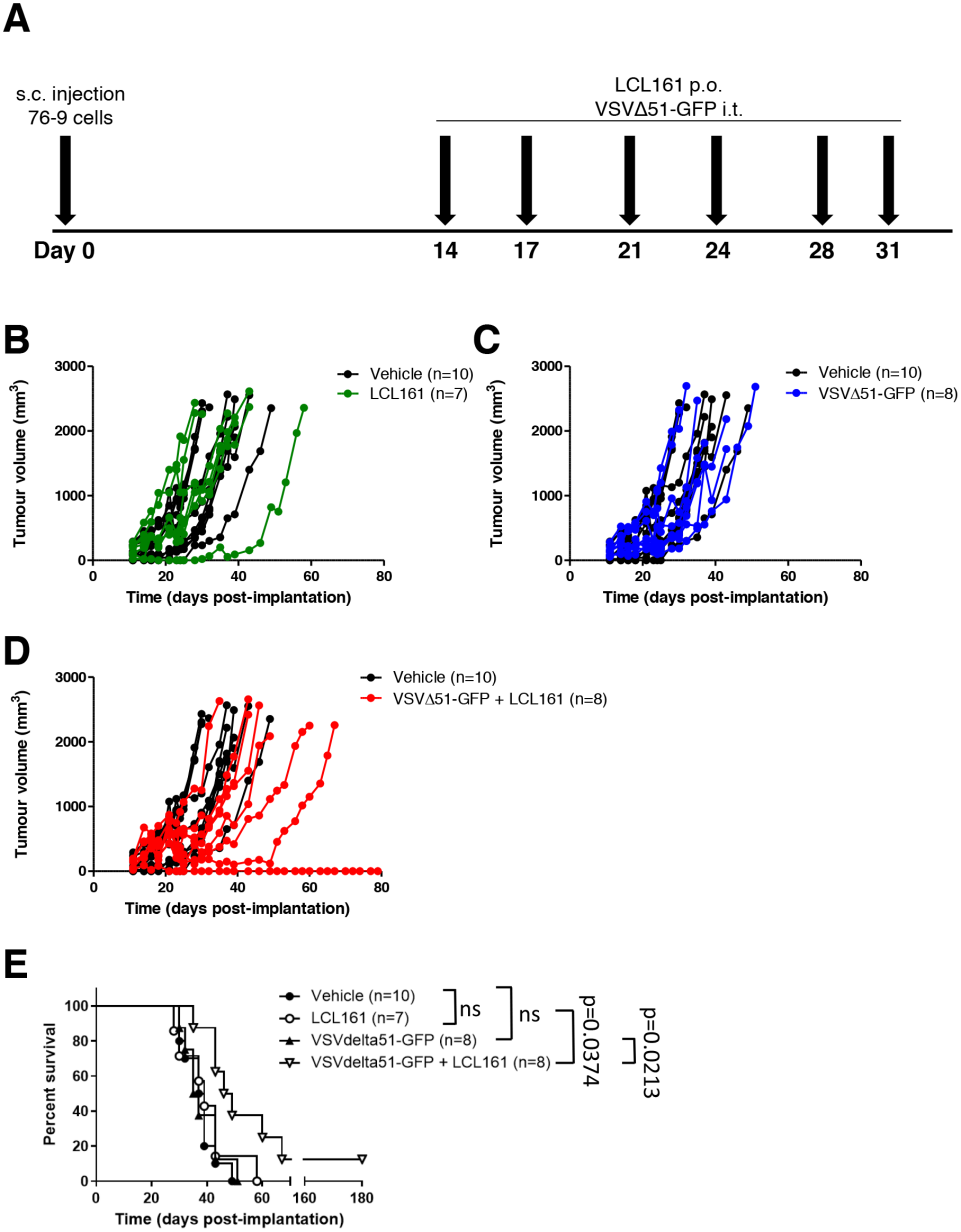
Combined therapy with LCL161 and VSVΔ51-GFP inhibits tumour growth in 76-9 RMS syngeneic mice **A**. Schematic of 76-9 tumour implantation and LCL161 and/or VSVΔ51-GFP treatment regimen. **B, D, F**. Female C57BL/6 mice with established 76-9 tumours were treated with vehicle, 50 mg/kg LCL161, 1 × 10^8^ pfu/ml of VSVΔ51-GFP, or LCL161 + VSVΔ51-GFP. Mice underwent 6 treatments in total and tumour growth was measured. **C, E, G**. Survival of the mice was monitored over time. The median survival was 38 days for vehicle treated-mice, 39 days for LCL161-treated mice, 36 days for VSVΔ51-GFP-treated mice, and 47.5 days for mice treated with a combination of LCL161 + VSVΔ51-GFP. (*p* < 0.05; Kaplan-Meier with log-rank analysis). (n = 7-10 mice per group).

To test the efficacy of combined LCL161 and VSVΔ51-GFP in a syngeneic tumour model *in vivo*, we treated female C57BL/6 mice bearing established 76-9 tumours with vehicle, 50 mg/kg LCL161, 1 × 10^8^ pfu/ml of VSVΔ51-GFP, or LCL161 + VSVΔ51-GFP. Mice were treated two times a week for three weeks (Figure 6A). Treatment with either LCL161 or VSVΔ51-GFP as a single agent did not affect tumour growth compared with vehicle-treated mice (Figure 6B and 6D). Similarly, the mean survival of LCL161-treated and VSVΔ51-GFP-treated 76-9 syngeneic mice was not different compared with vehicle-treated mice (Figure 6C and 6E). In contrast, combination treatment with LCL161 and VSVΔ51-GFP resulted in an overall reduction in the growth of 76-9 tumours compared with vehicle-treated mice (Figure 6F, p < 0.05). At 30 days post-implantation, tumour volume was 476.2 mm^3^ in mice that received combined treatment with LCL161 and VSVΔ51-GFP, whereas tumour volume was 1164.0 mm^3^ in vehicle-treated mice (*p* < 0.05). Kaplan-Meier survival curves with log-rank analysis determined that the median survival of 76-9 syngeneic mice treated with LCL161 and VSVΔ51-GFP was extended by 10 days compared with vehicle-treated mice (38 days vs. 47.5 days) with durable cures seen in 12.5% of the animals (Figure 6G, p < 0.05).

## DISCUSSION

RMS cancers are highly heterogeneous and are associated with a variety of genetic aberrations, including characteristic PAX3-FOXO1 or PAX7-FOXO1 fusion genes, as well as mutations in myogenic factors, cell cycle checkpoints, and growth signaling pathways [19]. Despite the heterogeneity of these cancers, we previously reported that the overexpression of two proteins, IGF2BP1 and cIAP1, occurs in all RMS patient-derived cell lines tested and in 75% of primary tumours [13]. The IAP proteins function as potent inhibitors of programmed cell death through the regulation of caspase activation and NF-κB signaling [8]. Therefore, we consider IAP antagonism by SMCs to be a promising target for therapeutic intervention for RMS cancers [8, 20]. Indeed, we previously reported that SMCs promote TNFα-mediated cell death in the RMS cell lines Kym-1, RH36, and RH41 and inhibit tumour growth in a Kym-1 xenograft model [13]. One goal of this study was to identify whether other RMS cell lines were sensitive to SMCs either as a single agent or in combination with alternative triggers of cell death pathways. Our results confirmed that only Kym-1 cells are sensitive to LCL161 as a single agent (Figure 1A). These results were expected since Kym-1 cells are the only RMS cell line tested thus far that has autocrine TNFα production. At concentrations up to 10μM, LCL161 did not reduce cell viability of any other RMS cell line, even though cIAP1 protein expression was reduced in all cell lines following 24 h incubation with LCL161 (Figure 1B). We have previously shown that Kym-1 cells are equally sensitive to cells death due to siRNA-mediated reduction of cIAP1 suggesting that the differences from other RMS cell lines are not due to off-target effects of SMCs in Kym-1 cells [13]. We also found that there is no difference in protein expression of apoptosis pathway effectors or immune modulator receptors in LCL161-resistant cells compared with LCL161-sensitive cells (Figure 1C and 1D), which eliminated the possibility of a single mechanism of resistance of RMS cell lines to IAP antagonism.

The current results, along with our previous report [13], suggest that RMS cancers may respond to combination therapy with SMCs and a pro-death ligand (e.g. TNFα) to promote the elimination of tumour cells. Since exogenous TNFα is highly toxic and is unlikely to be used as therapeutic agent, several other pro-death ligands have been recently investigated for combination use with SMCs. Indeed, combining SMCs and innate immune stimuli such as oncolytic viruses or TLR and death receptor agonists has been shown to be highly efficacious in treating multiple *in vitro* and *in vivo* cancer models and overcomes many of the limitations of either therapy used alone [8, 16, 21]. We hypothesized that we could exploit the immunomodulatory abilities of SMCs to specifically enhance the death of RMS cells when treated with stimulants of the immune system. Indeed, we showed *in vitro* that most human RMS cell lines are modestly sensitive to oncolytic virus as a single agent, but not other activators of innate immunity (IFNγ and TWEAK) (Figure 2). Kym-1 cells were highly sensitive to combination treatment with LCL161 and each of the activators of innate immunity we tested, further demonstrating the role of autocrine TNFα production in the killing of Kym-1 cells *in vitro*. In contrast, there was no additive or synergistic effect of LCL161 in combination with oncolytic virus to reduce cell viability in RH36 or RD cells. Interestingly, the combination of LCL161 and oncolytic virus resulted in synergistic killing of RH41 cells, although the high multiplicity of infection used in this *in vitro* experiment may limit the biological relevance of these findings. In order to better understand the mechanism of resistance of human RMS cell lines to LCL161 and activators of innate immunity, we measured TNFα and IRF1 mRNA levels following 24 h treatment with these compounds (Figure 3). TNFα mRNA expression was induced in Kym-1 and RH36 cells, but not RH41 cells, after treatment with oncolytic virus (Figures 3A-C). These data suggest that cell viability does not correlate with the induction of expression of TNFα in RMS cell lines following treatment with SMCs or activators of innate immunity. We also observed that IRF1 expression was induced by treatment with IFNγ in two out of three cell lines (Figures 3D-F). IRF1 is a nuclear transcription factor that is induced in response to IFNγ and plays an important role in inflammation, immunity, cell proliferation, and apoptosis [17]. It is hypothesized that the IFNγ-inducibility of IRF1 predicts better responsiveness to anti-cancer therapy [22]. However, the current results suggest that inducibility of mRNA expression of either TNFα or IRF1 does not predict the responsiveness of RMS cell lines to treatment with IAP antagonists or activators of innate immunity.

Another goal of this study was to determine the effect of combination therapy with SMC and activators of innate immunity in a mouse model of RMS. In order to do this, we performed *in vitro* studies examining the effect of LCL161 and activators of innate immunity on cell viability of the murine 76-9 RMS cell line. We confirmed that LCL161 synergizes with TNFα to reduce viability of 76-9 cells, but there was no effect of LCL161 in combination with IFNα, IFNβ, IFNγ, TRAIL, or TWEAK on cell viability (Figure 4). Similar to the human RMS cell lines, oncolytic virus was effective as a single agent to reduce viability in 76-9 cells, but there was no additive effect of LCL161 (Figure 5A and 5B). Treatment of 76-9 cells with oncolytic virus for 24 h also increased mRNA expression of TNFα (>190-fold increase), IRF1 (>9-fold increase), and IRF7. The transcription factor IRF7 plays a crucial role in the regulation of type I interferon response to viral infections [23], so we predicted the significant induction of IRF7 mRNA expression (>70-fold increase) in 76-9 cells that was observed following treatment with oncolytic virus. IRF7 mediates the production of cytokines in response to a viral infection and plays an important role in promoting apoptosis of virus-infected tumour cells [23, 24]. We further performed conditioned media experiments to demonstrate that treatment with VSVΔ51-GFP results in the production of secreted cytokines in 76-9 cells that are cytotoxic to neighbouring cells, although the observed effect was relatively modest (Figure 5F). In addition, we observed measurable TNFα protein in media from 76-9 cells infected with VSVΔ51-GFP, which supports previous reports that oncolytic viruses promotes cell killing via the NF-kB signaling pathway and TNFα activation [16, 25].

Finally, we tested the efficacy of combination therapy with SMC and oncolytic virus on the growth of RMS tumours in a syngeneic mouse model. There was no effect of LCL161 or oncolytic virus on 76-9 tumour volume when either was used as a single agent compared with vehicle-treated mice (Figure 6). Interestingly, the combination of LCL161 and oncolytic virus significantly reduced the growth of established 76-9 tumours and prolonged the survival of mice by 10 days compared with vehicle-treated mice, and afforded durable cure in 12.5% of the animals (Figure 6). Our findings are corroborated by several studies that have shown that SMCs and innate immune stimuli reduce tumour volume and prolong survival in murine tumour models [8, 16, 21, 26, 27]. Based on the discrepancy in our *in vitro* and *in vivo* data, we hypothesize that the effectiveness of this combination therapy relies on bystander killing *via* cytokines that are released from non-cancer cells in the tumour microenvironment [28]. It has been reported that oncolytic virus infections induce the secretion of cytokines and chemokines that kill cancer cells directly and promote bystander killing through the recruitment and activation of innate and adaptive immune cells that target the tumour [16, 29, 30]. Furthermore, cross-talk between cancer-associated fibroblasts and tumour cells is crucial for the success of oncolytic virus-based therapeutics and likely plays a role in tumour cell death [31]. Indeed, many of the most promising cancer immunotherapies that are currently under investigation are tailored to the tumour microenvironment [32]. Immune checkpoint inhibitors, including anti-cytotoxic T-lymphocyte antigen-4 (CTLA-4) and anti-programmed cell-death (PD-1), require recruitment of immune cells from the tumour microenvironment and have been shown to prevent tumour growth in various pre-clinical cancer models, including RMS and other sarcomas [33–35].

In summary, we demonstrate that combination immunotherapy involving SMCs and oncolytic virus can eradicate RMS tumours in a biologically relevant syngeneic murine model. Our study demonstrates the contrast between results obtained using *in vitro* versus *in vivo* experiments using IAP antagonists and innate immune stimuli and the important role of the tumour microenvironment (i.e. immune cells and non-tumour cells) in the success of cancer immunotherapies. Future studies will determine the mechanism of tumour cell targeting by this combined treatment paradigm and will promote its extension to human clinical trials. Future studies should also define the appropriate dose and timing of combinations of SMCs and immune triggers for RMS cancers. Furthermore, the importance of cytokine and chemokine production and the innate and adaptive immune response must be established in order to enhance the efficacy of anti-cancer immunotherapy treatment.

## MATERIALS AND METHODS

### Cell culture, reagents, and viruses

Human RMS cell lines (RH36, RH41, RD, RH18, RH28, and RH30) were a generous gift from Dr P Houghton (Department of Hematology-Oncology, St. Jude Children's Hospital, Memphis, TN, USA) and were cultured in RPMI 1640 complete media (supplemented with 10% heat-inactivated fetal calf serum (FCS), penicillin/streptomycin and L-glutamine). The human RMS cell line Kym-1 was purchased from the JCRB (Osaka, Japan) and cultured in Bulbecco's modified Eagle medium (DMEM)-F12 complete media. The murine RMS cell line 76-9 and the murine sarcoma cell line 1863 were a generous gift from Dr D Kirsch (Duke University Medical Center, Durham, NC, USA) and were cultured in DMEM complete media. The mouse myoblast cell line C2C12 (American Type Culture Collection, Manassas, VA, USA) was cultured in DMEM complete media. Primary human skeletal muscle myoblasts (HSMM; from post-gestational tissue, usually from the quadriceps or psoas tissue) were purchased and propagated as specified by the supplier (Lonza, Walkersville, MD, USA) and maintained in Clonetics SkGM-2 Bulletkit medium (Lonza). All cells were maintained at 37°C and 5% CO_2_.

The Smac mimetic reagent LCL161 was provided by Novartis [15, 36]. Recombinant TNFα and TRAIL were purchased from Enzo Life Sciences (Brockville, ON, Canada) an mouse universal type I interferon and IFN-β as well as human recombinant IFN-γ were purchased from PBL Assay Science (Piscataway, NJ, USA). Human and mouse recombinant TWEAK and mouse recombinant IFN-γ were purchased from R&D Systems (Minneapolis, MN, USA).

The Indiana serotype of VSVΔ51 was used in this study and was propagated in Vero cells [37]. Two derivatives of VSVΔ51 that have been previously characterized were used: a recombinant derivative of VSVΔ51 expressing green fluorescent protein (VSVΔ51-GFP) and a non-replicating VSVΔ51 with the deletion of the gene encoding for glycoprotein (VSVΔG-GFP) [16].

### Western blot analysis

Cells were scraped, collected by centrifugation, and lysed in radioimmunoprecipitation assay (RIPA) lysis buffer containing a protease inhibitor cocktail (Roche, Laval, QC, Canada). Equal amounts of soluble protein were separated on polyacrylamide gels followed by transfer to nitrocellulose membranes. Membranes were probed with antibodies against cIAP1/2 (CY-P1041) from CycLex (Nagano, Japan); caspase-3 (9661), caspase-8 (9746 and 4790) TWEAK Receptor/FN 14 (4403) from Cell Signaling Technologies (Danvers, MA, USA); TLR4 (sc-293072), FLIP (sc-5276), and IFNγRα (sc-700) from Santa Cruz Biotechnology (Dallas, TX, USA); c-FLIP (ADI-AAP-440) from Enzo LIFE Sciences (Farmingdale, NY, USA), XIAP (Cell Signalling), RIAP 3 ([38] to detect mouse XIAP) and tubulin (ab7291) from Abcam (Toronto, ON, Canada). Alexa Fluor 680 (Invitrogen, Burlington, ON, Canada) or IRDye800 (Li-Cor, Lincoln, NB, USA) were used to detect the primary antibodies, and infrared fluorescent signals were detected using the Odyssey Infrared Imaging System (Li-Cor).

### Quantitative RT-PCR analysis

qRT-PCR was performed as previously described using commercial primers from realtimeprimers.com or Qiagen [13]. Briefly, RNA was extracted from treated cells using RNAzol (Sigma-Aldrich, Oakville, ON, Canada) and 1 ug of total RNA used to make cDNA, using qScript cDNA Supermix (Quanta Biosciences, Gaithersburd, MD, USA). 0.5 or 1 µl of cDNA was then used to perform quantitative real time PCR using SSoAdvanced SYBR Green Supermix (Bio-rad, Mississauga, ON, Canada) for all mRNAs.

### *In vitro* viability assays

10,000 cells were seeded in 96-well plates and incubated overnight. Cells were treated with DMSO (vehicle) or LCL161 for 24 h and cell viability was determined by Alamar Blue (Resazurin sodium salt) assay (Sigma, Oakville, ON, Canada) with data were normalized to vehicle treatment. For immune stimulant experiments, 10 000 cells were seeded in 96-well plates, incubated overnight, and treated with DMSO or LCL161 and TWEAK, IFN-γ, IFN-β, universal type I IFN, or BSA (control) at the indicated concentrations for 24 h, followed by viability assays using Alamar Blue. For oncolytic virus viability assays, 10 000 cells were seeded in 96-well plates and the next day were treated with serial dilutions (10^3^ to 10^−6^ MOI) of VSVΔ51-GFP or VSVΔG-GFP in the presence of DMSO or LCL161 for 24 h or 48 h. Cell viability was assayed by Alamar Blue and data were normalized to vehicle treatment. Alternatively, cytotoxicity was determined using the CellPlayer™ cytotoxicity assay while the caspase 3 and 7 activity was measured using the IncuCyte™ Caspase-3/7 Apoptosis Assay Reagent and the IncuCyte™ ZOOM Content Kinetic Imaging System (Essen Bioscience) as described previously [13].

### Clonogenic assay

Long term survival was determined by clonogenic assay as described previously [16]. Briefly, 300,000 (RH36, RH41 and RD) or 350,000 (Kym-1) cells were seeded in 12-well plates and incubated overnight. Cells were treated with DMSO (vehicle) or LCL161 and BSA or VSVΔ51-GFP (MOI=0.01) for 24 h and then re-plated at 2000 cells per well in 6-well plates and incubated for 10 days. Subsequently, plates were washed 2x with PBS and fixed in 1ml of 3:1 Methanol:Acetic acid mix for 10 minutes. Wells were stained in 1 ml of 0.5% Crystal Violet in 50% Methanol for 10 minutes and after extensive rinsing were let dry at room temperature.

### Conditioned media experiments

600,000 76-9 cells were seeded in a 6-cm plate and incubated overnight. Cells were infected with VSVΔ51-GFP (MOI=0.01) for 24 h. Cell culture supernatant was centrifuged at 300 *x* g for 3 minutes and was exposed to UV light for 1 h to inactive viral particles. Subsequently, the UV-inactivated supernatant was applied to naive 76-9 cells in the presence of DMSO or 5 μM LCL161 for 48 h. Cell viability was assessed by Alamar Blue assay.

### Enzyme-linked immunosorbent assay (ELISA)

For TNF ELISA, Kym-1 (900,000 cells), RH36 (760,000 cells) or 76-9 cells (350,000 cells) were seeded in a 6-well plate for 24 h. Cells were treated with DMSO, PBS, BSA (negative controls) or 5 µM LCL161 and indicated immune stimulants (0.01 MOI of VSVΔ51-GFP; 100 ng/ml TWEAK) for 24h. Debris was removed by centrifugation and TNFα within the supernatant (76-9 cells) or cell lysate (Kym-1 and RH36) were measured using the mouse TNFα DuoSet ELISA kit (R&D Systems, Minneapolis, MN, USA) or human TNF ELISA kit (Thermo Scientific). Where required the samples were diluted 10X prior to ELISA in order to obtain detectable TNFα concentrations. For IRF1 ELISA, RH41 (750,000 cells) or RH36 (350,000) cells were seeded in a 6-well plate for 24 h. Cells were treated with DMSO, PBS, BSA (negative controls) or 5 µM LCL161 and indicated immune stimulants (0.01 MOI of VSVΔ51-GFP; 1000 units/ml IFN) for 24h. Debris was removed by centrifugation and total cell lysates were prepared in RIPA buffer. IRF1 levels were determined in undiluted lysates using the human IRF1 ELISA kit (Abbexa).

### 76-9 syngeneic murine model

4-5 week old, female C57BL/6 mice were implanted with 6 × 10^5^ 76-9 cells by subcutaneous injection into the right hind flank and tumours were grown until palpable (~100 mm^3^). Mice were randomly assigned to treatment group and each group had a minimum of 7 mice for statistical measures. Mice were treated with 1 × 10^8^ pfu/ml of virus (i.t.) early morning followed by vehicle (30% 0.1 m HCl, 70% 0.1 m NaOAc (pH 4.63)) or 50 mg/kg LCL161 *per os* in the late afternoon on post-implantation days 14, 17, 21, 24, 28, and 31 (6 treatments total). Tumour size and body weight was measured three times weekly over the course of the study. Tumour volume was calculated using (π)(W)^2^(L)/4, where W = tumour width and L = tumour length. Animal technicians were blinded to treatment groups and euthanized mice when tumour burden exceeded 2,500 mm^3^. All animal experiments were conducted with the approval of the University of Ottawa Animal Care and Veterinary Service in concordance with guidelines established by the Canadian Council on Animal Care.

### Statistical analysis

The data are presented as group mean ± s.e.m. and were analyzed using GraphPad Prism 5 (GraphPad Software Inc., San Diego, CA, USA). Unless otherwise noted, all results were obtained through a minimum of three independent experimental replications. Cell viability was analyzed by two-way repeated-measures analysis of variance (ANOVA), followed by *post hoc* analysis for a statistically significant F-statistic (*p* <0.05) using Student's *t*-test with Bonferroni correction for multiple comparisons. For RT-qPCR experiments, average RNA expression was calculated using data collected from three biological replicates and three technical replicates for each biological replicate. Tumour volume in mice was analyzed by two-way repeated-measures ANOVA (independent variables: treatment and post-implantation day). Survival was assessed by Kaplan-Meier curves with log-rank analysis. Two groups of data were considered to be statistically different when *p* < 0.05.

## SUPPLEMENTARY MATERIALS FIGURES


