# Supplementary material for: Oncolytic virus synergizes with Smac mimetic compounds to induce rhabdomyosarcoma cell death in a syngeneic murine model
Source: Oncotarget. 2016 Dec 10;8(2):3495–508. doi: 10.18632/oncotarget.13849 (PMC5356898; doi:10.18632/oncotarget.13849)
Supplement: Supplementary file 1 [file oncotarget-08-3495-s001.pdf]

Oncolytic virus synergizes with Smac mimetic compounds to induce rhabdomyosarcoma cell death in a syngeneic murine model

SUPPLEMENTARY FIGURES

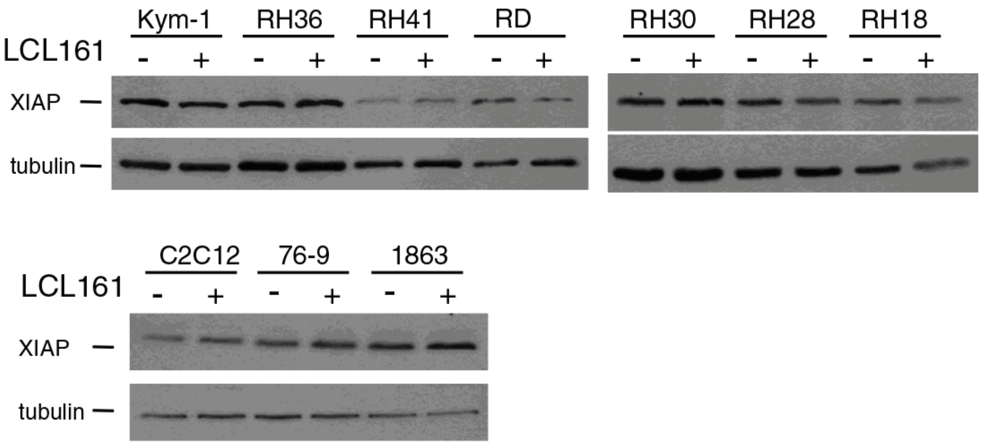

Supplementary Figure S1: Human RMS cell lines were analysed by western blot for XIAP (anti-XIAP, Cell Signalling) and tubulin protein expression following treatment with vehicle control (DMSO) or 5 µM LCL161 (10 nM LCL161 for Kym-1 cells) for 24 h. The mouse C2C12 myoblast, 76-9 RMS, and 1863 sarcoma cell lines were also analysed by western blot for XIAP (Anti-RIAP3, [38]) and tubulin protein expression following treatment with DMSO or 5 µM LCL161 for 24 h.

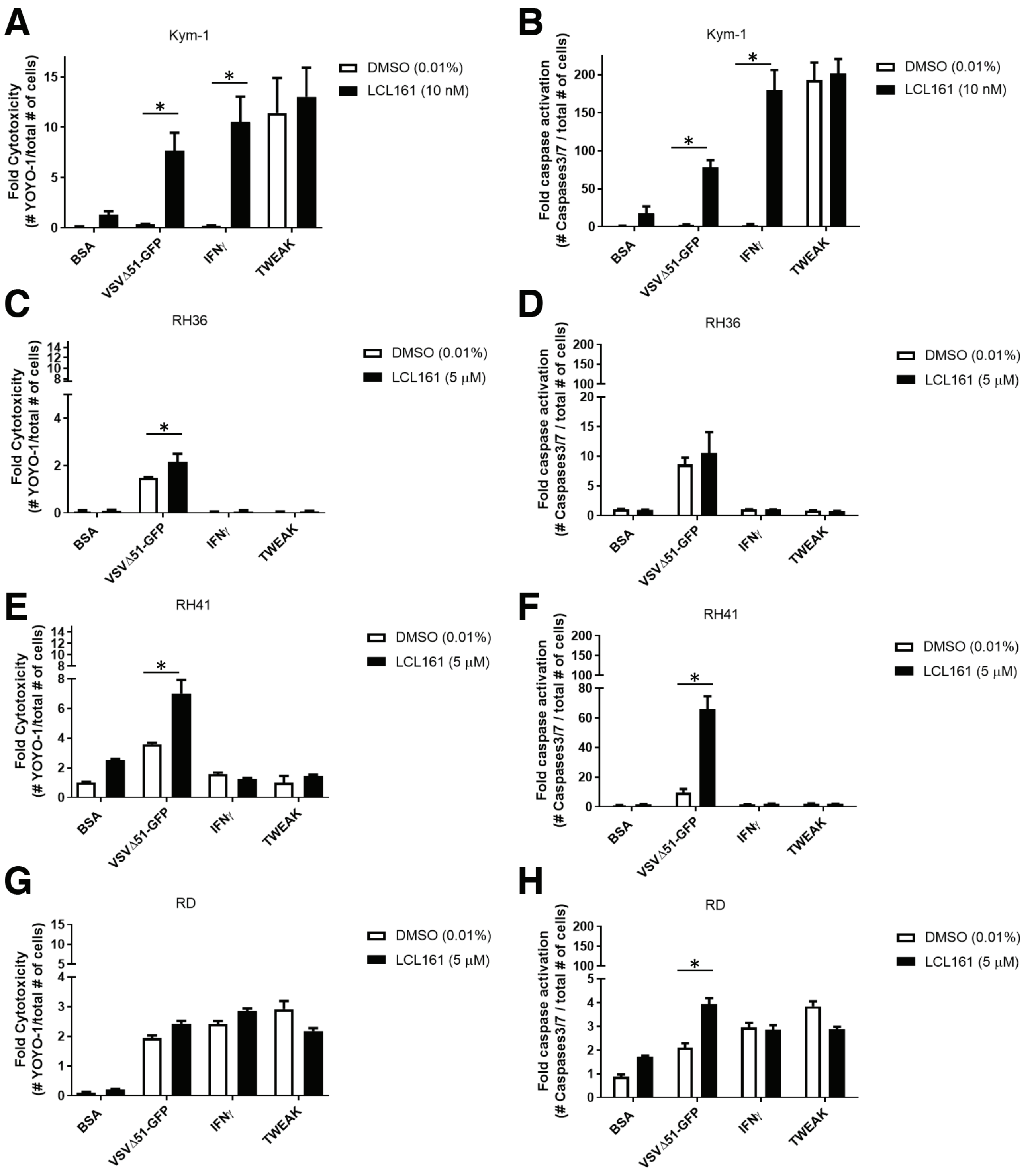

Supplementary Figure S2: Cell viability **A, C, E, G** and caspase 3/7 activity **B, D, F, H** of human RMS cell lines treated with vehicle control (DMSO) or 5 μM LCL161 (10 nM LCL161 for Kym-1) and indicated immune stimulants for 24 hr was determined by the CellPlayer™ and the IncuCyte™ Caspase-3/7 Apoptosis Assay Reagent, respectively, and the IncuCyte™ ZOOM Content Kinetic Imaging System.

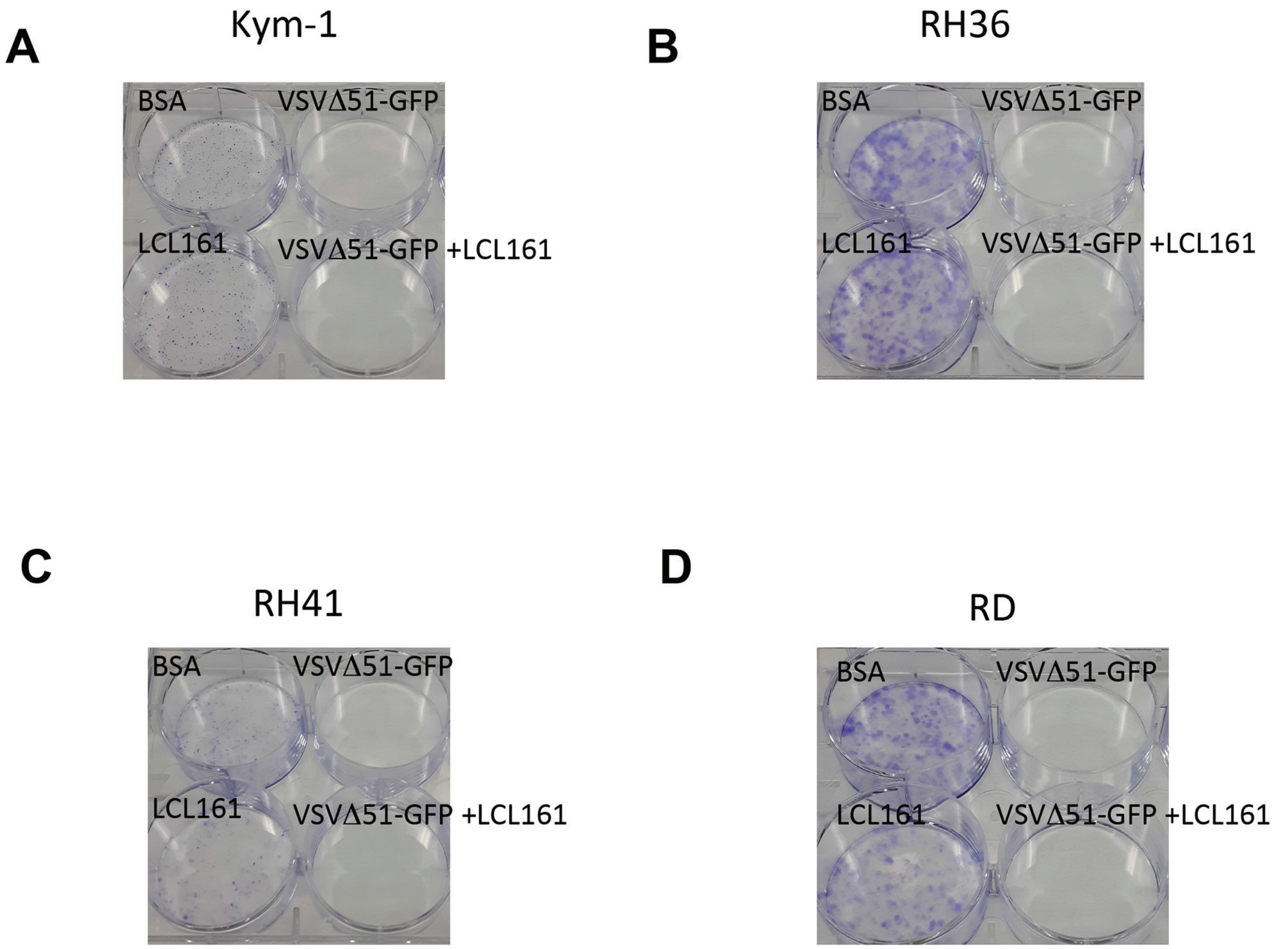

Supplementary Figure S3: Long term survival of human RMS cells treated with vehicle control (DMSO) or 5 µM LCL161 (10 nM LCL161 for Kym-1 cells) and VSVΔ51-GFP was determined by the clonogenic assay.

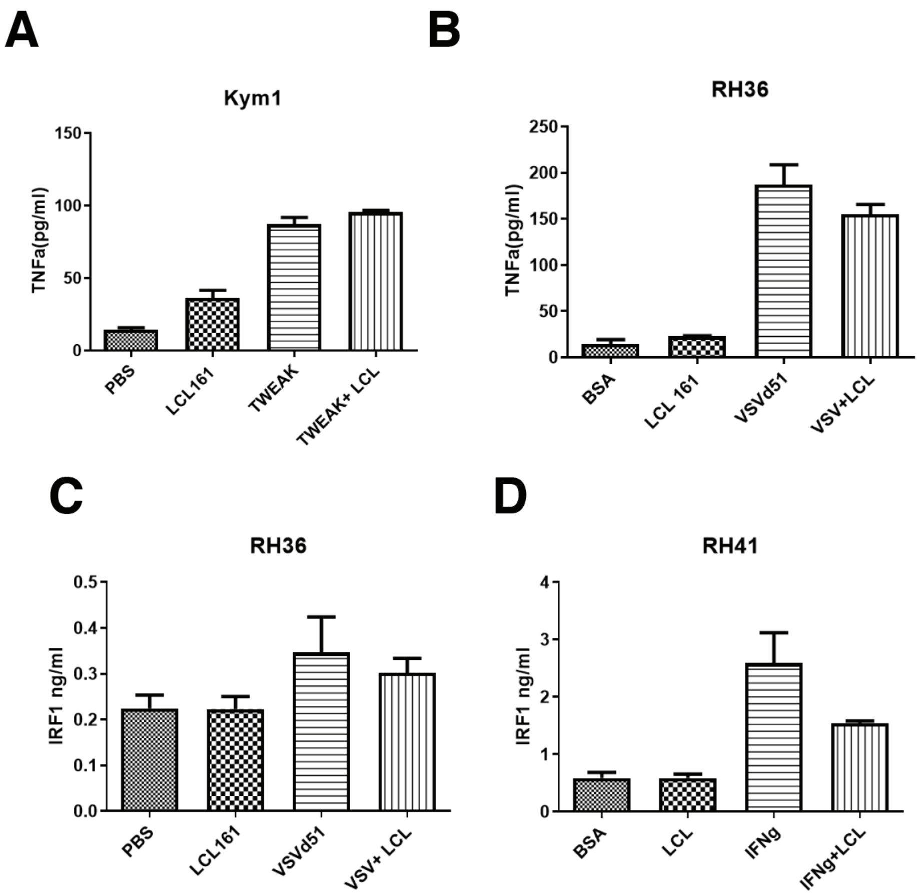

Supplementary Figure S4: The levels of TNFα and IRF1 proteins in RMS cells treated with vehicle control (DMSO) or 5 µM LCL161 (10 nM LCL161 for Kym-1 cells) and VSVΔ351-GFP (0.01 MOI), TWEAK (100 ng/ml) or IFNγ (1000 units/ml) was determined by ELISA.
